# Factors Affecting Mortality in Elderly Patients Hospitalized for Nonmalignant Reasons

**DOI:** 10.1155/2014/584315

**Published:** 2014-08-03

**Authors:** Teslime Ayaz, Serap Baydur Sahin, Osman Zikrullah Sahin, Ozlem Bilir, Halil Rakıcı

**Affiliations:** ^1^Department of Internal Medicine, Faculty of Medicine, Recep Tayyip Erdogan University, Rize 53100, Turkey; ^2^Department of Endocrinology, Faculty of Medicine, Recep Tayyip Erdogan University, Rize 53100, Turkey; ^3^Department of Nephrology, Faculty of Medicine, Recep Tayyip Erdogan University, Rize 53100, Turkey; ^4^Department of Emergency, Faculty of Medicine, Recep Tayyip Erdogan University, Rize 53100, Turkey; ^5^Department of Gastroenterology, Faculty of Medicine, Recep Tayyip Erdogan University, Rize 53100, Turkey

## Abstract

Elderly population is hospitalized more frequently than young people, and they suffer from more severe diseases that are difficult to diagnose and treat. The present study aimed to investigate the factors affecting mortality in elderly patients hospitalized for nonmalignant reasons. Demographic data, reason for hospitalization, comorbidities, duration of hospital stay, and results of routine blood testing at the time of first hospitalization were obtained from the hospital records of the patients, who were over 65 years of age and hospitalized primarily for nonmalignant reasons. The mean age of 1012 patients included in the study was 77.8 ± 7.6. The most common reason for hospitalization was diabetes mellitus (18.3%). Of the patients, 90.3% had at least a single comorbidity. Whilst 927 (91.6%) of the hospitalized patients were discharged, 85 (8.4%) died. Comparison of the characteristics of the discharged and dead groups revealed that the dead group was older and had higher rates of poor general status and comorbidity. Differences were observed between the discharged and dead groups in most of the laboratory parameters. Hypoalbuminemia, hypertriglyceridemia, hypopotassemia, hypernatremia, hyperuricemia, and high TSH level were the predictors of mortality. In order to meet the health necessities of the elderly population, it is necessary to well define the patient profiles and to identify the risk factors.

## 1. Introduction

Life expectancy increases each year due to the improvements in living conditions and advances in medicine resulting in an incremental increase in elderly population [1, 2]. Physiological, psychological, and social problems of the elderly require different approaches and this necessitates the physicians being ready for the increasing population of the elderly. Many factors including aging-associated diseases, chronic diseases and comorbidities, multiple drug use, functional disability, and feeding problems make health care difficult in the elderly. In addition, symptoms and signs vary in the elderly and are usually atypical [2]. It is known that physiological processes show variations in the elderly as compared to young individuals; many biomarkers alter with age, and within this context, laboratory reference range values also differ in elderly population [3, 4].

Elderly population is hospitalized more frequently and for a longer period of time as compared to young population and they suffer from more severe diseases that are difficult to diagnose and treat [5]. Patients aged 65 years or over have been reported to be hospitalized approximately 3 times more frequently than young individuals [6]. Additionally, it has been reported that one-third of them are discharged with lower functional capacity than they admitted with and that approximately 5% die during hospital stay, whereas 20%–30% die within one year following hospital discharge [6]. It has been reported that some known traditional risk factors causing poor prognosis may vary in the elderly and even some may act opposite to the expected effect. In a study conducted in a very elderly population, low body mass index (BMI), low diastolic blood pressure, and low cholesterol levels (which indicate reverse metabolic syndrome) were found to be significant predictors of mortality [7]. In the light of this information, comprehensive studies are needed to better understand the factors that influence mortality in elderly population.

The present study aimed to investigate the factors affecting mortality in elderly patients hospitalized for nonmalignant reasons and to determine the relation between laboratory parameters measured routinely in clinical practice and in-hospital mortality, as well as to determine the predictors of mortality.

## 2. Methods

This is a retrospective cohort study of hospitalized patients followed during hospitalization.

Records of the patients hospitalized in the Internal Medicine Clinic of our hospital between September 2010 and September 2012 were retrospectively reviewed. Patients over the age of 65 years and hospitalized primarily for nonmalignant reasons were included in the study. Surgery or trauma patients were excluded. Study participants comprised also the patients who had comorbid malignant diseases, as well as the patients treated for malignancy who were cured or in remission. Demographic data, reasons for hospitalization, comorbidities, duration of hospital stay, and results of the routine blood tests at the time of first hospitalization were obtained from the hospital records. The study was conducted in accordance with the ethical rules for human experimentation that are stated in the Declaration of Helsinki. These inpatients' privacy and confidentiality are guaranteed.

### 2.1. Statistical Analysis

Data were analyzed using the Statistical Package for the Social Sciences (SPSS, Inc., Chicago, IL, USA) version 15.0 for Windows program. Descriptive statistics were expressed as cross-tables for categorical variables and as mean ± standard deviation for numerical variables. Independent categorical variables were compared using chi-square test. In the event that chi-square assumption was not met, Fisher's exact test was used for paired group comparison. Paired group comparisons were performed using independent samples *t*-test for normally distributed numerical variables or using Mann-Whitney *U* test for nonnormally distributed numerical variables. Risk factors were identified by backward method and logistic regression analysis. A *P* value of <0.05 was considered statistically significant.

## 3. Results

The present study included 1012 patients, of whom 592 were female with a mean age of 78.4 ± 7.8 years and 420 were male with a mean age of 77.0 ± 7.1 years. The most common reason for hospitalization was diabetes mellitus (DM), followed by poor general status and kidney diseases. Of the patients, 90.3% had at least a single comorbidity. The most frequently observed comorbidities were hypertension and cardiovascular diseases. Demographic characteristics of the patients and information about hospitalization are summarized in Table 1.

Whilst 927 (91.6%) of the hospitalized patients were discharged from the hospital (discharged group), 85 (8.4%) died (dead group).

Evaluation of the general characteristics of the discharged and dead groups revealed that the dead group was older and that poor general status at the time of hospitalization, comorbidities of DM, malignant disease and cirrhosis, and presence of at least a single comorbidity were significantly more prevalent in this group (Table 2).

Comparison of the discharged and dead groups in terms of laboratory results revealed that the mean values of platelets, iron, total iron-binding capacity (TIBC), albumin, calcium, total cholesterol, low-density lipoprotein (LDL) cholesterol, and high-density lipoprotein (HDL) cholesterol were significantly lower in the dead group. The mean values of mean platelet volume (MPV), erythrocyte sedimentation rate (ESR), urea, uric acid, creatinine, alanine aminotransferase (ALT), aspartate aminotransferase (AST), sodium, triglyceride, and C-reactive protein (CRP) were significantly higher in the dead group (Table 3).

In the discharged and dead groups, the rates of patients with normal laboratory values were compared with those of patients with values lower or higher than the normal laboratory values. The rates of patients with a low hemoglobin level, thrombocytopenia, a high ESR, a low TIBC, uremia, hyperuricemia, hypercreatininemia, hypoalbuminemia, a high AST level, a high ALT level, hypernatremia, hypopotassemia, hyperpotassemia, hypocalcemia, hypertriglyceridemia, a low HDL level, a low thyroid stimulating hormone (TSH) level, a high CRP level, and a positive HBsAg were significantly higher in the dead group. The rate of patients with hypercholesterolemia and the rate of those with a high LDL were significantly lower in the dead group (Table 4).

In order to identify the risk factors for mortality, analysis was performed in a model including the following variables: gender, age, poor general status, renal disease diabetes mellitus, cardiovascular disease, pulmonary disease, gastrointestinal disease, infections, anemia, rheumatologic disease and electrolyte imbalance as the reason for hospitalization, presence of DM, hypertension, cardiovascular disease, malignancy, chronic renal failure and cirrhosis as comorbidities, duration of hospital stay, CRP, HBsAg, anti-HCV antibody, anti-HIV antibody, hemoglobin, platelet, MPV, ESR, iron, TIBC, MCV, glucose, urea, uric acid, creatinine, albumin, ALT, AST, sodium, potassium, calcium, cholesterol, triglyceride, HDL cholesterol, LDL cholesterol, and TSH. Poor general status as the reason for hospitalization, presence of malignancy or cirrhosis as comorbidity, hypoalbuminemia, hypertriglyceridemia, hypopotassemia, hypernatremia, hyperuricemia, and high TSH were found to be the significant risk factors for mortality (Table 5).

## 4. Discussion

In the present study, the rate of in-hospital mortality was found to be 8.4% in the hospitalized patients over 65 years of age. To the best of our knowledge, there are very few studies performed on this subject in elderly patients. In addition, different patient populations having different reasons for hospitalization, differences in the lower age limit of the participants, and differences in duration of hospital stay require attention when comparing the results. The rate of in-hospital mortality among elderly patients has been reported to be 12% [8], 14.9% [9], and 16.4% [10] in various studies.

In addition to common parameters, different risk factors have also been reported in the limited number of studies investigating general risk factors for mortality in the hospitalized elderly. Ponzetto et al. [9] investigated the risk factors affecting mortality in 987 hospitalized patients aged ≥70 years and determined functional impairment, medical condition-related disability, cerebrovascular disease, cancer, an albumin level of 3–3.4 g/dL, a creatinine level of 1.5–3 mg/dL, a creatinine level of >3 mg/dL, and a fibrinogen level of ≥452 mg/dL as the independent risk factors for in-hospital mortality. Sousa et al. [8] evaluated 158 hospitalized patients aged >70 years and found a subjective medical evaluation at the beginning of the hospital admission, a low Barthel score, a low serum albumin, and a high white blood cell (WBC) count to be correlated with high mortality. Silva et al. [10] evaluated 856 patients aged between 60 and 104 years and determined that delirium, cancer, immobility, low albumin levels, elevated creatinine levels, history of heart failure, and advanced age were associated with increased mortality. In a study conducted to investigate known cardiovascular risk factors that influence mortality in elderly population, Zhang et al. [11] found that left ventricular ejection fraction (LVEF), LDL and HDL cholesterol levels, albumin level, and creatinine clearance rate (CCR) were significantly lower and the incidence of atrial fibrillation was higher in the patients with all-cause mortality than in the survived patients. Moreover, they reported LVEF, atrial fibrillation, LDL cholesterol, albumin, and CCR to be significant and independent predictors for mortality in multivariate Cox regression model. Buurman et al. [12] evaluated 639 hospitalized patients aged ≥65 years in terms of risks for functional decline; of the patients, 27% were at low risk, 33% were at intermediate risk, and 40% were at high risk for developing new disability. High risk patients were also reported to be at high risk for mortality. Fontana et al. [13] assessed the relation between 17 biomarkers and mortality in 594 hospitalized elderly patients. They reported blood levels of insulin-like growth factor (IGF-1), triiodothyronine (T3), CRP, ESR, WBC and lymphocyte counts, iron, albumin, total cholesterol, and LDL cholesterol to be associated with mortality. They also determined in multivariate Cox proportional hazard model that IGF-1, CRP, hemoglobin, and glucose were the biomarkers that most strongly predicted the mortality. Moreover, studies have reported malnutrition [14], red blood cell distribution width (RDW) [15], advanced age (≥85 years) [16], medical adverse events [17], high serum vitamin B12 level [18], and arrhythmia [19] among the parameters associated with mortality in hospitalized elderly patients.

In the present study, comparison of the characteristics of the discharged and dead groups demonstrated that general condition was poor and comorbidity was higher in the dead group, and laboratory parameters varied between the two patient groups. In the risk factor analysis, poor general status as the reason for hospitalization, presence of malignancy or cirrhosis as comorbidity, hypoalbuminemia, hypertriglyceridemia, hypopotassemia, hypernatremia, and a high TSH level were determined to be the predictors of mortality.

Among the risk factors affecting mortality, the presence of comorbidity was 89.6% in the discharged group, whereas it was significantly higher in the dead group with a rate of 97.6%. The most common comorbidities were cardiovascular diseases, DM, and hypertension. Ahluwalia et al. [20] determined the prevalence of comorbidity to be higher in the hospitalized patients than in the nonhospitalized patients in a population aged over 65 years and having heart failure (*n* = 201130) and found that comorbidity was associated with an increased risk for mortality in the whole population. They reported myocardial infarction, DM, chronic kidney disease, chronic obstructive pulmonary disease, dementia, depression, hip fracture, stroke, colorectal cancer, and lung cancer to be significantly associated with the risk for mortality in hospitalized patients. In the present study, malignancy (OR = 4.381) and cirrhosis (OR = 9.628) were the comorbidities determined as risk factors for mortality.

Hypoglycemia can occur during hospital stay regardless of the presence of DM. Kagansky et al. [21] reported that hypoglycemia was determined at a rate of 5.2% in the hospitalized patients over the age of 70 years and that mortality was two times higher in the hypoglycemic patients than in the nonhypoglycemic patients. Nevertheless, in the same study, sepsis, a low albumin level, and malignancy, but not hypoglycemia, were found to be independent predictors of mortality by multivariate analysis. In the present study, hyperglycemia and hypoglycemia were determined in about 60% and 10% of the patients, respectively; there was no difference between the discharged and dead groups in this respect.

A low albumin level has been associated with mortality in the hospitalized elderly patients [8, 10, 11, 18, 21]. In the present study, hypoalbuminemia was more frequent in the dead group (89.4 versus 50.4), and it was also found to be a significant risk factor (OR = 3.818).

Electrolyte imbalance, mainly due to DM and diuretics, is a common problem in elderly population. The frequency of mild electrolyte disorder has been reported to be 15% in the general population aged over 55 years [22]. Electrolyte imbalance is one of the conditions necessary to be monitored closely because of its life-threatening character [22]. Hyponatremia and hypernatremia are among the most frequently encountered electrolyte disorders in hospitalized patients and are associated with increased mortality [23]. In a study in which sodium concentration was measured in 3182 patients (mean age, 53 ± 21 years) admitted to the emergency room, hyponatremia and hypernatremia were determined at the rates of 4% and 13%, respectively, and the patients with hyponatremia were reported to be older [24]. In another study, hyponatremia was determined in 14.5% of 98411 adult hospitalized patients, hyponatremic patients were reported to be older and to have more comorbidity, and hyponatremia was found to be associated with increased in-hospital mortality [25]. In the present study, we also found the frequency of hypernatremia to be significantly higher in the dead group than in the discharged group (33.3% versus 6.1%). Although the frequency of hyponatremia was higher in the dead group, the difference was not statistically significant as compared to the discharged group (34.4% versus 28.4%). In the present study, hypernatremia was found effective on mortality in the risk factor analysis (OR = 3.5).

Severe hyperkalemia (hyperpotassemia) is known to be a life-threatening electrolyte imbalance. Hyperkalemia in hospitalized patients is encountered at a rate between 1% and 10% [26]. In a study conducted in 923 adult patients with severe hyperkalemia, the rate of in-hospital mortality was found to be 30.7% despite appropriate and aggressive treatment. In addition to the contribution of comorbidities to the increased mortality, hyperkalemia itself has been reported to have a role [27]. In the present study, the rates of hyperpotassemia (32.9% versus 20.4%) and hypopotassemia (24.2% versus 9.7%) were significantly higher in the dead group as compared to the discharged group. Nevertheless, in the risk factor analysis, only hypopotassemia was found to be a significant risk factor (OR = 2.582).

Studies have demonstrated an association between thyroid hormones and mortality in elderly population [28, 29]. De Alfieri et al. [30] investigated thyroid hormone as a predictor of mortality in the hospitalized very old patients (mean age, 84 years). They emphasized that low T3 syndrome contributed to in-hospital mortality; however, hormone levels were influenced by comorbidities, drug interactions, and nutritional status; therefore, the results should be cautiously interpreted. Iglesias et al. [31] investigated the relation between in-hospital mortality and thyroid functions in 447 patients aged >60 years and reported alterations in thyroid function tests in 74.3% of the patients as well as a positive association between the presence of these alterations and the age of the patients and in-hospital mortality. They also reported the decrease in free-T3 value to be a powerful predictor of in-hospital mortality in elderly patients. In the present study, the rates of both low TSH (39.2% versus 18.9%) and high TSH levels (11.1% versus 4.1%) were higher in the dead group as compared to the discharged group. The risk factor analysis revealed that high TSH significantly influenced the mortality (OR = 6.166).

In conclusion, in order to meet the health necessities of the elderly population, which is gradually increasing worldwide, it is necessary to well define the patient profiles and to identify the risk factors, and further studies are required to collect more data on this subject.

## Figures and Tables

**Table 1:** General characteristics of the patients.

Characteristics	
Age, years	77.8 ± 7.6
Gender	
Female	592 (58.5)
Male	420 (41.5)
Reason for hospitalization	
Poor general status	169 (16.7)
Diabetes mellitus	185 (18.3)
Cardiovascular diseases	67 (6.6)
Pulmonary diseases	96 (9.5)
Gastrointestinal diseases	95 (9.4)
Renal diseases	157 (15.5)
Infection	77 (7.6)
Anemia	124 (12.3)
Rheumatologic diseases	9 (0.9)
Electrolyte imbalance	25 (2.5)
Other	8 (0.8)
Presence of at least one comorbidity	914 (90.3)
Comorbidity	
Cardiovascular disease	412 (40.7)
Hypertension	610 (60.3)
Diabetes mellitus	389 (38.4)
Chronic renal failure	65 (6.4)
Malignant diseases	91 (9.0)
Cirrhosis	23 (2.3)
Other	200 (19.8)
Duration of hospital stay, days	7.7 ± 5.9

Values are presented as mean ± standard deviation or number (%), where appropriate.

**Table 2:** General characteristics of the discharged and dead groups.

Characteristics	Discharged group (*n* = 927)	Dead group (*n* = 85)	*P*
Age, years	77.6 ± 7.5	80.7 ± 7.7	**<0.001**
Gender			
Female	540 (58.3)	52 (61.2)	0.601
Male	387 (41.7)	33 (38.8)	
Reason for hospitalization			
Poor general status	114 (12.3)	55 (64.7)	**<0.001**
Diabetes mellitus	182 (19.6)	3 (3.5)	—
Cardiovascular diseases	64 (6.9)	3 (3.5)	—
Pulmonary diseases	93 (10.0)	3 (3.5)	—
Gastrointestinal diseases	90 (9.7)	5 (5.9)	—
Renal diseases	147 (15.9)	10 (11.8)	0.318
Infection	75 (8.1)	2 (2.4)	—
Anemia	121 (13.1)	3 (3.5)	—
Rheumatologic diseases	9 (1.0)	0 (0.0)	—
Electrolyte imbalance	24 (2.6)	1 (1.2)	—
Other	8 (0.9)	0 (0.0)	—
Presence of at least one comorbidity	831 (89.6)	83 (97.6)	**0.017**
Comorbidity			
Cardiovascular disease	371 (40.0)	41 (48.2)	0.140
Hypertension	555 (59.9)	55 (64.7)	0.383
Diabetes mellitus	346 (37.3)	43 (50.6)	**0.016**
Chronic renal failure	62 (6.7)	3 (3.5)	0.256
Malignant diseases	68 (7.3)	23 (27.1)	**<0.001**
Cirrhosis	18 (1.9)	5 (5.9)	**0.037**
Other	188 (20.3)	12 (14.1)	0.172
Duration of hospital stay, days	7.7 ± 5.8	8.0 ± 6.7	0.449

**Table 3:** Laboratory values of the discharged and dead groups at the time of hospitalization.

Characteristics	Discharged group (*n* = 927)	Dead group (*n* = 85)	*P*
Hemoglobin (g/dL)	11.3 ± 2.2	10.9 ± 1.9	0.069
MCV (fL)	85.0 ± 8.0	84.9 ± 7.3	0.964
PLT (K/*µ*L)	267.9 ± 123.4	239.9 ± 144.1	**0.002**
MPV (fL)	8.5 ± 1.7	9.1 ± 2.2	**0.009**
ESR (mm/h)	48.1 ± 34.2	61.0 ± 37.5	**0.001**
Iron (*µ*g/dL)	46.3 ± 42.1	35.9 ± 31.7	**0.003**
TIBC (*µ*g/dL)	197.8 ± 93.7	152.3 ± 89.3	**<0.001**
Glucose (mg/dL)	159.7 ± 103.5	167.6 ± 135.4	0.905
Urea (mg/dL)	84.1 ± 57.8	127.7 ± 75.5	**<0.001**
Uric acid (mg/dL)	6.6 ± 2.7	9.1 ± 3.9	**<0.001**
Creatinine (mg/dL)	2.0 ± 1.9	2.4 ± 1.8	**0.010**
Albumin (g/dL)	3.4 ± 0.6	2.7 ± 0.6	**<0.001**
ALT (U/L)	27.0 ± 66.8	43.8 ± 107.3	**0.027**
AST (U/L)	30.1 ± 53.7	66.7 ± 130.4	**0.001**
Sodium (mmol/L)	137.9 ± 6.4	140.4 ± 9.6	**0.022**
Potassium (mmol/L)	4.5 ± 0.8	4.5 ± 1.1	0.313
Calcium (mg/dL)	8.8 ± 0.9	8.3 ± 1.0	**<0.001**
Triglyceride (mg/dL)	134.1 ± 73.1	155.0 ± 89.8	**0.045**
Total cholesterol (mg/dL)	170.9 ± 49.5	147.5 ± 62.5	**<0.001**
LDL cholesterol (mg/dL)	105.7 ± 38.7	91.0 ± 53.4	**<0.001**
HDL cholesterol (mg/dL)	36.0 ± 13.8	25.2 ± 16.1	**<0.001**
TSH (*µ*IU/mL)	1.5 ± 3.2	2.1 ± 5.0	0.196
CRP (mg/dL)	6.1 ± 8.2	10.6 ± 10.9	**<0.001**

MCV: mean corpuscular volume; PLT: platelet; MPV: mean platelet volume; ESR: erythrocyte sedimentation rate; TIBC: total iron-binding capacity; ALT: alanine aminotransferase; AST: aspartate aminotransferase; LDL: low-density lipoprotein; HDL: high-density lipoprotein; TSH: thyroid stimulating hormone; CRP: C-reactive protein.

**Table 4:** Abnormal laboratory results at the time of hospitalization in the discharged and dead groups.

Problem	Definition	Discharged group (*n* = 927)	Dead group (*n* = 85)	*P*
Low hemoglobin	Hb < 12.2 g/dL	593 (64.0)	65 (76.5)	**0.021**
Thrombocytopenia	PLT < 142 K/*µ*L	90 (10.7)	18 (22.5)	**0.002**
High ESR	ESR E: > 20 K: > 30 mm/h	614 (66.3)	69 (81.2)	**0.005**
Low TIBC	<120 *µ*g/dL	182 (19.8)	31 (36.5)	**<0.001**
Uremia	Urea > 43 mg/dL	687 (74.4)	79 (92.9)	**<0.001**
Hyperuricemia	Uric acid > 6 mg/dL	490 (54.4)	65 (78.3)	**<0.001**
Hypercreatininemia	Creatinine > 1.1 mg/dL	506 (56.3)	59 (72.0)	**0.006**
Hypoalbuminemia	Albumin < 3.5 mg/dL	466 (50.4)	76 (89.4)	**<0.001**
High ALT	ALT > 55 U/L	56 (6.0)	13 (15.3)	**0.001**
High AST	AST > 34 U/L	180 (19.4)	29 (34.5)	**0.001**
Hypernatremia	Sodium > 145 mmol/L	41 (6.1)	21 (33.3)	**<0.001**
Hypopotassemia	Potassium < 3.5 mmol/L	73 (9.7)	15 (24.2)	**<0.001**
Hyperpotassemia	Potassium > 5.1 mmol/L	174 (20.4)	23 (32.9)	**0.014**
Hypocalcemia	Calcium < 8.4 mg/dL	269 (29.9)	41 (49.4)	**<0.001**
Hypertriglyceridemia	Triglyceride > 149 mg/dL	292 (31.5)	41 (48.2)	**0.002**
Hypercholesterolemia	Total cholesterol > 199 mg/dL	228 (24.6)	12 (14.1)	**0.030**
High LDL cholesterol	LDL > 100 mg/dL	492 (53.1)	31 (36.5)	**0.003**
Low HDL cholesterol	HDL < 35 mg/dL	424 (46.4)	64 (76.2)	**<0.001**
Low TSH	TSH < 0.35 *µ*IU/mL	169 (18.9)	31 (39.2)	**<0.001**
High TSH	TSH > 4.94 *µ*IU/mL	31 (4.1)	6 (11.1)	**0.030**
High CRP	CRP > 0.8 mg/dL	639 (68.9)	70 (82.4)	**0.010**
HBsAg positivity		11 (1.2)	4 (4.7)	**0.031**

Hb: hemoglobin; PLT: platelet; ESR: erythrocyte sedimentation rate; TIBC: total iron-binding capacity; ALT: alanine aminotransferase; AST: aspartate aminotransferase; LDL: low-density lipoprotein; HDL: high-density lipoprotein; TSH: thyroid stimulating hormone; CRP: C-reactive protein.

**Table 5:** Risk factor analysis for mortality.

	*P*	OR	95.0% CI	
			Lower bound	Upper bound
Poor general status as the reason for hospitalization	**<**0.001*	7.964	4.435	14.299
Presence of malignancy as comorbidity	**<**0.001*	4.381	2.179	8.810
Presence of cirrhosis as comorbidity	0.001*	9.628	2.653	34.938
Low hemoglobin	0.078	1.825	0.934	3.564
Uremia	0.060	2.658	0.959	7.366
Hypoalbuminemia	0.001*	3.818	1.699	8.583
High ALT	0.066	2.128	0.952	4.756
Sodium (normal)	0.001	1		
Hyponatremia	0.235	0.677	0.355	1.290
Hypernatremia	0.002*	3.500	1.596	7.675
Potassium (normal)	0.038	1		
Hypopotassemia	0.018*	2.582	1.178	5.658
Hyperpotassemia	0.144	1.631	0.846	3.146
Uric acid (normal)	0.040	1		
Hypouricemia	0.421	1.988	0.374	10.573
Hyperuricemia	0.011*	2.366	1.216	4.605
Hypertriglyceridemia	0.020*	1.935	1.108	3.381
TSH (normal)	0.005	1		
Low TSH	0.485	1.249	0.670	2.328
High TSH	0.001*	6.166	2.048	18.568

OR: odds ratio; CI: confidence interval; ALT: alanine aminotransferase; TSH: thyroid stimulating hormone.

*P < 0.05 is statistically significant.
